# Antiretroviral treatment associated hyperglycemia and dyslipidemia among HIV infected patients at Burayu Health Center, Addis Ababa, Ethiopia: a cross-sectional comparative study

**DOI:** 10.1186/1756-0500-7-380

**Published:** 2014-06-21

**Authors:** Molla Abebe, Samuel Kinde, Getachew Belay, Atsbeha Gebreegziabxier, Feyissa Challa, Tefera Gebeyehu, Paulos Nigussie, Belete Tegbaru

**Affiliations:** 1 Department of Clinical Chemistry, School of Biomedical and Laboratory Sciences, College of Medicine and Health Sciences, University of Gondar, Gondar, Ethiopia; 2 Department of Medical Laboratory Sciences, School of Allied Health Sciences, College of Health Sciences, Addis Ababa University, Addis Ababa, Ethiopia; 3 Ethiopian Health and Nutrition Research Institute, Addis Ababa, Ethiopia

**Keywords:** HIV/AIDS, Dyslipidemia, Hyperglycemia, Antiretroviral therapy, Ethiopia

## Abstract

**Background:**

The effects of highly active antiretroviral therapy (HAART) on glucose and lipid metabolism among sub-Saharan Africans, for whom access to antiretroviral therapy is expanding, remain largely unknown. Therefore, the aim of this study was to assess antiretroviral treatment associated hyperglycemia and dyslipidemia among HIV infected patients at Burayu health center, Addis Ababa, Ethiopia.

**Methods:**

A cross-sectional comparative study was conducted among HIV infected adults at Burayu Health Center, Addis Ababa, Ethiopia from September, 2011 to May, 2012. Equal number of HAART naïve and HAART initiated patients (n = 126 each) were included in the study. Demographic data were collected using a well-structured questionnaire. Total cholesterol (TC), Triglyceride (TG), high density lipoprotein cholesterol (HDL-C), low density lipoprotein cholesterol (LDL-C) and glucose were determined. The data were analyzed using SPSS version 20 software.

**Result:**

Of 252 study participants, 72.2% were females; mean age was 35.3 years; mean Body Mass Index (BMI) was 21.4(kg/m^2^); mean time living with the virus was 20.6 months and 15.5% were TB-HIV co-infected. The prevalence of hyperglycemia, increased LDL-C hypercholesterolemia, hypertriglyceridemia and decreased HDL-C were 7.9%, 23%, 42.1%, 46.8% and 50.8% in HAART and 5.6%, 7.1%, 11.1%, 31% and 73% in non-HAART groups, respectively. First line antiretrovirals were drugs containing 2 nucleoside backbones (from Zidovudine/Stavudine/Lamivudine/Tenofovir) with either Nevirapine or Efavirenz. There was statistically significant increase in serum lipid profile levels among HAART initiated patients than HAART naïve individuals (p =0.01 for TG and <0.001 for others).

**Conclusion:**

First-line HAART is associated with potentially atherogenic lipid profile levels in patients with HIV infection compared to untreated patients. This indicates glucose and lipid profile levels need to be monitored regularly in HIV infected patients taking antiretroviral treatment.

## Background

Highly active antiretroviral therapy (HAART) is the mainstay of treatment for those infected with HIV [1]. Since its introduction in 1996, mortality and morbidity rates in HIV-infected individuals in countries with widespread access to HAART have plummeted. The main effect of HAART is to suppress viral replication, allowing the individual’s immune system to recover and protecting from the development of AIDS and death [2]. In recent years, provision of HAART to those in need has become an increasingly important and feasible global priority [3]. However, the prospect of maintaining patients on long term HAART may be restricted by a heterogeneous collection of unexpected metabolic abnormalities, including dysregulation of glucose metabolism, dyslipidemia, and/or lipodystrophy [4,5]. Use of HAART has been linked to hyperglycemia, dyslipidemia and increased risk of cardiovascular disease (CVD) in HIV-infected patients in industrialized countries. The effects of HAART on glucose and lipid metabolism among sub-Saharan Africans, for whom access to antiretroviral therapy is expanding, remain largely unknown [6]. This is specially a major gap that should be given high emphasis in a county like Ethiopia where increased use of HAART is higher since 2005. Therefore, the aim of this study was to assess antiretroviral treatment associated hyperglycemia and dyslipidemia among HIV infected patients at Burayu health center, Addis Ababa, Ethiopia.

## Methods

### Study design, setting and period

A cross-sectional comparative study was conducted at voluntary counseling and testing (VCT) center of Burayu Health Center in collaboration with Ethiopian Health and Nutrition Research Institute (EHNRI) from September, 2011 to May, 2012. Two groups of study participants with age ≥18 years who were either on HAART for at least six months (HAART initiated group) and HAART naïve HIV patients (non-HAART groups) who visited the VCT center during the study period were included in the study. First-line HAART regimens used were nucleoside reverse transcriptase inhibitors (NRTIs) [lamivudine (3TC), zidovudine (AZT), stavudine (D4T) or Tenofovir (TDF)] and non-nucleoside reverse transcriptase inhibitors (NNRTIs) [nevirapine (NVP) or efavirenz (EFV)]. Patients with hyperglycemia or dyslipidemia at baseline (for HAART initiated patients) and pregnant women and nursing mother were excluded.

### Data collection

Consecutive sampling technique was employed to include study participants. Two hundred fifty two (126 HAART initiated and 126 non-HAART) participants were enrolled. After having received a clear explanation of the objective, risk and confidentiality of the study, participants signed the informed consent and participated in the study. Demographic data regarding, age, gender, period with HIV infection, ART exposure in the previous six months and the ARV regimen were collected using questionnaire. Medical records were also used to confirm the information and baseline glucose and lipid profile levels. Height and weight were measured to calculate body mass index (BMI). Six to ten ml of blood sample was collected by venipuncture from 8–12 hours fasting individuals using vacuum tube (Becton Dickinson, SA) and serum was separated within one hour of blood collection. The serum levels of glucose, TC, HDL-C, LDL-C and TG were measured using COBAS INTEGRA 400 random access full automated auto analyzer at EHNRI, clinical chemistry laboratory. Glucose was determined by Hexokinase (HK) method, TG and total TC were evaluated with enzymatic colorimetric method and HDL-C and LDL-C were analyzed by homogenous enzymatic colorimetric method. CD4+ lymphocyte count was determined using flow cytometer (FACSCalibur, Becton Dickinson, CA, USA). Tuberculosis (TB) was diagnosed via microscopy from sputum sample and X-ray photography if smear negative TB encountered.

The cutoff points to categorize dyslipidemia was defined as TC ≥200 mg/dl, HDL-c <40 mg/dl, LDL-c ≥130 mg/dl, TG ≥150 mg/dl and hyperglycemia as glucose ≥110 mg/dl and were based on the United States National Cholesterol Education Program, Adult Treatment Panel (NCEP-ATP) III guideline [7].

### Data analysis

Data were entered and analyzed using Statistical Package for Social Sciences (SPSS) window version 20 (IBM Statistics, USA). Chi-square, student-t-test and logistic regression tests were used to see the association between socio-demographic characteristics, duration with HIV, BMI and HAART regimens with serum glucose and lipid profile levels. P-Value <0.05 was considered to be statistically significant at 95% confidence interval (CI).

### Ethical consideration

The study was ethically cleared from the Departmental Research and Ethics Review Committee of Department of Medical Laboratory Sciences, College of Health Sciences, Addis Ababa University, Oromia Regional Health Bureau Ethical Review Committee, EHNRI Scientific and Ethical Review Committee and National Research Ethics committee of Ethiopia. VCT center counselors (nurses from the Health Center) who collected patient data were oriented and trained to keep data confidentiality. Patients received their results through clinicians for further diagnosis and treatment accordingly.

## Results

### Socio demographic characteristics of study subjects

A total of 252 HIV infected patients were enrolled in the study. Of these, 126 (50%) were initiated HAART (HAART group) and 126 (50%) were ART naive (non-HAART group). Majority of the study participants were females (182 (72.2%)). Of those HAART groups, 86 (68.3%) were females. In addition, among non-HAART groups, 96 (76.2%) were females. The overall mean age was 35.3 ± 10.2 years (range: 18 to 75 years). The total time from the serological diagnosis of HIV infection was 20.6 ± 17.3 months (Mean ± SD) and was longer in HAART group compared to non-HAART group (mean: 29.5 ± 17.1 versus 11.8 ± 12.3 months, p <0.0001). Thirty four (27%) HAART groups and 25 (19.8%) non-HAART groups had CD4+ cells <200 cells/mm^3^. Of all the subjects, 39 (15.5%) had TB-HIV co infection during the study period [21(16.7%) of the HAART and 18 (14.3%) non-HAART groups] (Table 1).

**Table 1:** Characteristics of study population by HAART status at Burayu Health center, Addis Ababa, Ethiopia, 2012

**Variable**		**HAART initiated (n=126)**	**Non-HAART (n=126)**
		**N (%)**	**N (%)**
Gender	Female	86(68.3)	96(76.2)
	Male	40(31.7)	30(23.8)
Age group (years)	Mean ± SD	37.1(9.8)	33.4(10.2)
	18-29	25(19.8)	51(40.5)**
	30-39	61(48.4)	49(38.9)
	40-49	25(19.8)	16(12.7)
	≥50	15(11.9)	10(7.9)
BMI	Mean ± SD	21.9(3.2)	20.8(2.8)
	<18.5	9(7.1)	24(19)*
	18.5-24.9	97(77)	91(72.2)
	25-29.9	17(13.5)	10(7.9)
	≥30	3(2.4)	1(0.8)
CD4+ cells (cells/mm^3^)	Mean ± SD	314.5 ± 152.5	413.6 ± 246.2
	<200	34(27)	25(19.8)**
	200-400	58(46)	42(33.3)
	>400	34(27)	59(46.8)
Duration of HIV infection (month)	Mean ± SD	29.5(17)	11.8(12.3)
	<6	0(0)	57(45.2)***
	6-12	22(17.5)	21(16.7)
	13-24	22(17.5)	27(21.4)
	25-41	59(46.8)	19(15.1)
	≥42	23(18.3)	2(1.6)
TB Co-infection	Present	21(16.7)	18(14.3)
	Not present	105(83.3)	108(85.7)

N, number; BMI, body mass index; HAART, highly active antiretroviral therapy; TB, tuberculosis; SD, standard deviation; *p-value =0.02; **p-value =0.004; ***p-value <0.0001.

### Characteristics of serum fasting glucose and lipid profile levels

Totally, 17 (6.7%) individuals had abnormally high fasting serum glucose level. There was no significant difference in serum glucose level between HAART initiated and non-HAART groups (P =0.45). However, there was higher serum lipid profile levels among HAART groups than HAART naïve individuals (p =0.01 for TG and <0.001 for others) (Table 2).

**Table 2:** Serum lipid profile and glucose levels of study population by HAART status at Burayu Health Center, Addis Ababa, Ethiopia, 2012

**Parameters**		**HAART initiated (n=126)**	**Non-HAART (n=126)**
		**N (%)**	**N (%)**
Glucose	Mean ± SD	97.1 ± 15.7	92.2 ± 16.9*
	≤110 mg/dl	116(92.1)	119(94.4)
	>110 mg/dl	10(7.9)	7(5.6)
Triglycerides	Mean ± SD	162.2 ± 92.7	131.5 ± 55.3**
	<150 mg/dl	67(53.2)	87(69)
	≥150 mg/dl	59(46.8)	39(31)*
Total cholesterol	Mean ± SD	194.3 ± 45.2	147.2 ± 37.7***
	<200 mg/dl	73(57.9)	112(88.9)
	≥200 mg/dl	53(42.1)	14(11.1)***
HDL-cholesterol	Mean ± SD	40.9 ± 12.9	32.6 ± 11.3***
	<40 mg/dl	64(50.8)	92(73)***
	≥40 mg/dl	62(49.2)	34(27)
LDL-cholesterol	Mean ± SD	107.2 ± 35	81.4 ± 29.3***
	<130 mg/dl	97(77)	117(92.9)
	≥130 mg/dl	29(23)	9(7.1)***

HAART, highly active antiretroviral therapy; HDL, high-density lipoprotein; LDL, low- density lipoprotein; SD, standard deviation; *p-value =0.01; **p-value =0.002; *** p-value <0.0001.

### Serum fasting glucose/lipid profile level and HAART

Most of the HAART initiated patients (43, 34.1%) were on ART for 13–24 months followed by those for 25–41 months (42, 33.3%). The rest 28 (22.2%) and 13 (10.3%) were on ART for 6–12 months and above 3.5 years, respectively. Duration of HAART was not significantly associated with serum glucose/lipid profile levels.

**Figure 1:**
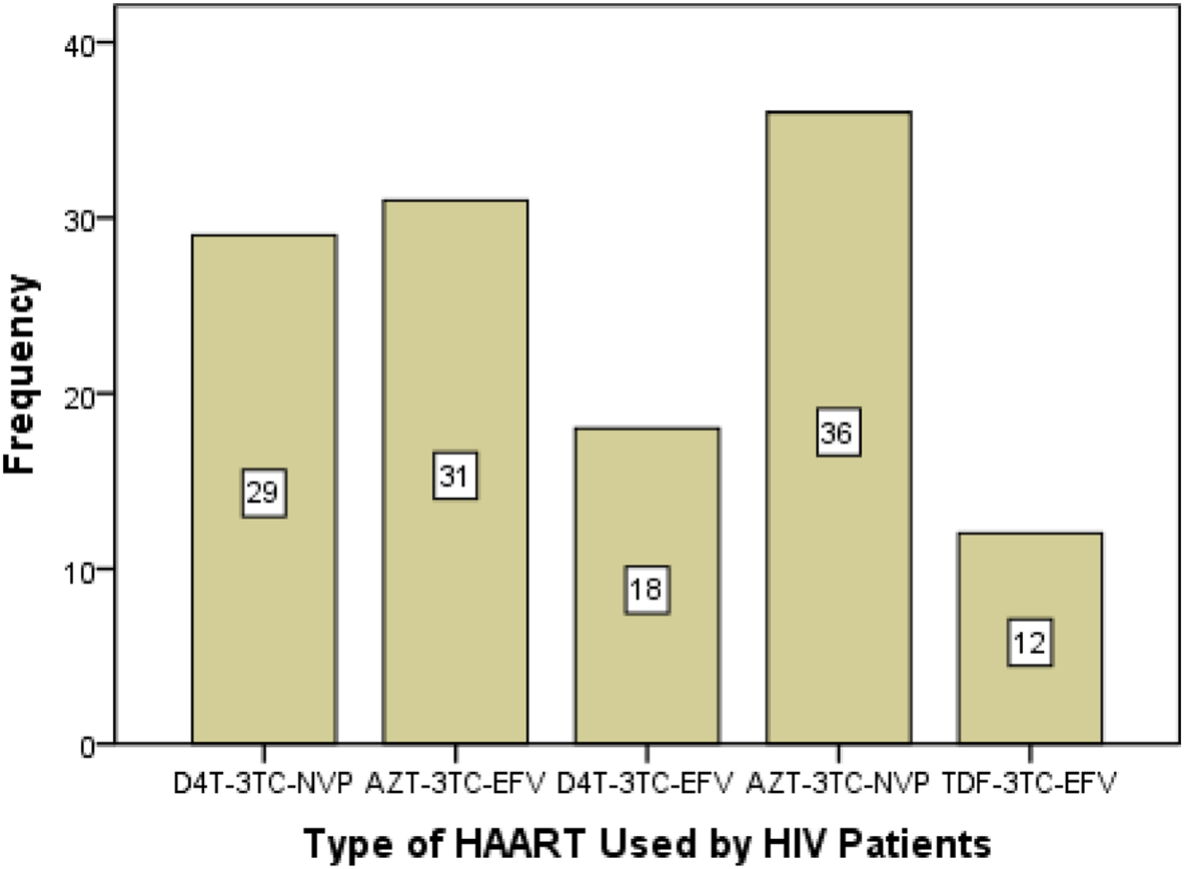
Frequency of different combinations of antiretroviral drugs used by HIV infected patients at Burayu Health Center, Addis Ababa, Ethiopia, 2012.

A total of 4 nucleoside reverse transcriptase inhibitors [Lamivudine (3TC), Zidovudine (AZT), Stavudine (D4T) and Tenofovir (TDF)] and 2 non-nucleoside reverse transcriptase inhibitors [Nevirapine (NVP) and Efavirenz (EFV) with 5 combinations (D4T-3TC-NVP, AZT-3TC-EFV, D4T-3TC-EFV, AZT-3TC-NVP and TDF-3TC-EFV] were used to treat patients. Nevirapine and EFV based combinations were used by almost equivalent number of individuals [65(51.6%) versus 61(48.4%), respectively]. In addition, D4T and AZT based ARVs were used by 47 (37.3%) and 67 (53.2%) individuals, respectively. AZT-3TC-NVP combinations of antiretrovirals were the most frequently used drugs among the HAART initiated study participants, [36 (28.6%)] while TDF-3TC-EFV combinations were relatively used by small number of patients, [12 (9.5%)] (Figure 1). No significant difference observed in glucose/lipid profile derangements between patients receiving AZT based combinations compared to those on D4T; and patients treated with EFV based combinations versus those treated with NVP (Table 3).

**Table 3:** Serum glucose and lipid profile levels distribution among different combinations of HAART taken by study participants at Burayu Health Center, Addis Ababa, Ethiopia, 2012

**Glucose/lipid profile**		**NVP versus EFV based combination of ARVs**		**P-value**
		**NVP (n=65)**	**EFV (n=61)**	
		**N (%)**	**N (%)**	
Glucose	≤110	60(92.3)	56(91.8)	0.917
	>110	5(7.7)	5(8.2)	
Triglyceride	<150	37(56.9)	30(49.2)	0.384
	≥150	28(43.1)	31(50.8)	
Total cholesterol	<200	37(56.9)	36(59)	0.812
	≥200	28(43.1)	25(41)	
HDL-cholesterol	<40	28(43.1)	36(59)	0.074
	≥40	37(56.9)	25(41)	
LDL-cholesterol	<130	48(73.8)	49(80.3)	0.388
	≥130	17(26.2)	12(19.7)	
**Glucose/lipid profile**		**D4T versus AZT based combination of ARVs**		**P-value**
		**D4T (n=47)**	**AZT (n=67)**	
		**N (%)**	**N (%)**	
Glucose	≤110	40(85.1)	64(95.5)	0.053
	>110	7(14.9)	3(4.5)	
Triglyceride	<130	25(53.2)	35(52.2)	0.920
	≥130	22(46.8)	32(47.8)	
Total cholesterol	<200	28(59.6)	39(58.2)	0.884
	≥200	19(40.4)	28(41.8)	
HDL-cholesterol	<40	26(55.3)	33(49.3)	0.524
	≥40	21(44.7)	34(50.7)	
LDL-cholesterol	<130	37(78.7)	53(79.1)	0.961
	≥130	10(21.3)	14(20.9)	

AZT, Zidovudine; d4T, stavudine; EFV, efavirenz; NVP, nevirapine; p, significance level.

### Serum glucose/lipid profile levels and risk factors

Using univariate analysis, there was no statistically significant association between serum glucose/lipid profile levels and gender. However, there were statistically significant association between TG and HAART initiation, TC and HAART initiation, HDL-C and HAART initiation, and LDL-C and HAART initiation (p = 0.01, <0.0001, < 0.0001 and 0.001, respectively). Glucose was not affected by different variables except by CD4+cells (p =0.023). Similarly, tuberculosis (TB) co-infection slightly affected serum LDL-C level only (p =0.49) (Table 4).

**Table 4:** Association of glucose and lipid profile levels and other variables at Burayu Health Center, Addis Ababa, Ethiopia, 2012

**Variables**	**Glucose/lipid profile levels**				
	**Gluc >110 mg/dl**	**TG ≥ 150 mg/dl**	**TC ≥ 200 mg/dl**	**HDL < 40 mg/dl**	**LDL ≥ 130 mg/dl**
	**UOR (95% CI)**	**UOR (95% CI)**	**UOR (95% CI)**	**UOR (95% CI)**	**UOR (95% CI)**
Gender (Female)	0.52(0.19-1.43)	0.62(0.36-1.10)	1.18(0.63-2.23)	1.37(0.77-2.45)	1.85(0.77-4.42)
P-value	0.208	0.097	0.608	0.289	0.167
Age ≥35years	1.52(0.56-4.12)	1.23(0.78-2.14)	2.30(1.29-4.10)	1.38(0.83-2.30)	2.57(1.23-5.35)
P-value	0.414	0.329	0.005	0.217	0.012
BMI >21.2	0.94(0.35-2.53)	1.27(0.76-2.11)	2.21(1.24-3.92)	1.78(1.07-2.98)	1.78(0.88-3.60)
P-value	0.908	0.358	0.007	0.028	0.108
CD4 + cells <200/mm^3^	3.21(1.18-8.73)	1.59(0.88-2.86)	0.82(0.42-1.62)	0.96(0.52-1.75)	0.57(0.23-1.44)
P-value	0.023	0.124	0.570	0.884	0.233
HAART Initiated	1.47(0.54-3.98)	1.96(1.17-3.29)	5.81(3.01-11.2)	2.62(1.55-4.44)	3.89(1.76-8.61)
P-value	0.453	0.01	<0.0001	< 0.0001	0.001
HIV duration ≥20 months	1.12(0.42-2.99)	1.19(0.72-1.98)	1.98(1.11-3.51)	1.37(0.82-2.28)	2.11(1.03-4.35)
P-value	0.828	0.500	0.020	0.231	0.042
TB infection	1.19(0.32-4.33)	1.11(0.56-2.23)	0.80(0.36-1.79)	0.68(0.33-1.42)	2.28(1.00-5.18)
P-value	0.798	0.590	0.590	0.307	0.049

HAART, highly active antiretroviral therapy; Gluc, glucose; UOR, un adjusted odds ratio; CI, confidence interval; BMI, body mass index; TC, total cholesterol; HDL, high-density lipoprotein cholesterol; LDL, low-density lipoprotein cholesterol; TG, triglyceride; p, significance level; TB, tuberculosis.

Adjusting for potential confounding factors such as gender, age, BMI, CD4 + cells, duration with HIV, TB co-infection and HAART initiation, HAART initiation and BMI had an independent and positive association with raised serum TC level. In addition, HAART initiation was independently associated with serum HDL-C and LDL-C levels (Table 5).

**Table 5:** Association between cholesterol and HAART initiation, adjusted for the potential confounding factors of study participants at Burayu Health Center, Addis Ababa, Ethiopia, 2012

**Variables**	**Lipid profile levels**		
	**TC ≥200 mg/dl**	**HDL < 40 mg/dl**	**LDL ≥130 mg/dl**
	**AOR (95% CI)**	**AOR (95% CI)**	**AOR (95% CI)**
Non-HAART*	1.00	1.00	1.00
HAART Initiated	5.46(2.81-10.61)	2.62(1.55-4.44)	3.89(1.76-8.61)
P-value	<0.0001	<0.0001	0.001
BMI ≤21.2*	1.00	1.00	1.00
BMI >21.2	1.90(1.03-3.50)		
P-value	0.039		

HAART, highly active antiretroviral therapy; AOR, adjusted odds ratio; CI, confidence interval; BMI, body mass index; TC, total cholesterol; HDL, high-density lipoprotein cholesterol; LDL, low-density lipoprotein cholesterol; p, significance level; *Reference category.

## Discussion

In this study, we tried to address how presence and absences of HAART affects glucose and lipid profile levels in HIV positive individuals.

Our results showed an increased prevalence of hyperglycemia, hypercholesterolemia and hypertriglyceridemia in patients receiving ARV drugs than non-HAART study participants. The prevalence of hyperglycemia (>110 mg/dl), low density lipoprotein cholesterol (LDL-C) hypercholesterolemia (≥130 mg/dl), total cholesterol (TC) hypercholesterolemia (≥200 mg/dl), hypertriglyceridemia (≥150 mg/dl) and high density lipoprotein cholesterol (HDL-C) hypocholesterolemia (<40 mg/dl) were 7.9%, 23%, 42.1%, 46.8% and 50.8% in HAART initiated and 5.6%, 7.1%, 11.1%, 31% and 73% in non-HAART groups, respectively.

In this study, we found comparable percentage of HAART initiated individuals (7.9%) and non- HAART group (5.6%) who had an increased serum glucose level and HAART initiation was not associated (p =0.45). However, HAART group showed higher mean glucose level than non-HAART group (p =0.019). In agreement to this finding, a study in Brazil observed elevated glucose levels in 6.8% (7/103) patients treated without PIs, 1.5% (2/134) patients receiving PIs and 0.9% (1/112) non-HAART individuals despite the small number cases limited their conclusions [8].

Among our study subjects, there was no association between serum glucose/lipid level and gender. However, according to a study from Thailand on 200 HAART treated patients for an average of 39.35 months, the prevalence of hyperlipidemia was higher in men than women despite no difference in blood glucose level between the two. Among those reported patients, 6.5%, 10.5%, 34.0% and 35.5% developed increased LDL-C, diabetes mellitus, hypercholesterolemia and hypertriglyceridemia, respectively [9]. The discrepancy with our study may be due to our study participants were not gender matched between the two groups.

Increased serum lipid profile level was associated with HAART initiation (p = 0.01 for TG and <0.0001 for others) in our study. Similar to our findings, a cross-sectional study from India showed significantly higher prevalence of dyslipidemia in the first line treatment groups [10]. Moreover, according to a study from the same country, at baseline and at 12 months, TC was >200 mg/dl for 1% and 26% of patients; LDL-C level was >130 mg/ dl for 3% and 23%; HDL-C level was <40 mg/dl for 91% and 23% and blood glucose level was >110 mg/dl for 14% and 13%, respectively [11].

In Canada, among 745 ARV treated patients, 10% and 16% showed increased TG and TC levels, respectively [12]. Unlike to the above and in agreement with ours a study from Cameroon showed the prevalence of TC ≥200 mg/dl as 37.6% and 24.6%, respectively in ART groups and ART naive groups (p =0.019). The equivalents for LDL-C ≥130 mg/dl were 46.4% and 21% (p ≤0.001) [13].

Inline to our finding, a long term analysis on plasma lipid concentration was performed in patients starting first-line antiretroviral therapy in Netherlands and showed concentrations of TC, LDL-C and TG continued to increase with slight decrease in HDL-C [14]. Similarly, a study from Cameroon showed TC, LDL-C and HDL-C levels increased significantly (P <0.05) but TG remained unaltered with first line ARV therapy for 3 months [15]. This might be due to short treatment period.

In our HAART initiated study participants, the mean serum HDL-C, LDL-C, TG and TC levels were 40.9, 107.2, 162.2 and 194.3 mg/dl, respectively. The result of another 1 year follow up study in Italy showed elevated mean levels of serum HDL-C, 40.1 mg/dl; LDL-C, 165.2 mg/dl; TC, 258.7 mg/dl and TG, 306.4 mg/dl than ours. These mean differences might be probably due to population difference in Ethiopia and Italy. They found decreased HDL-C level 9.4%, hypercholesterolemia 25%, increased LDL-C level 26.7% and hypertriglyceridemia 38.2% [16] which was almost comparable with our results. Moreover, a 5 year cohort study in Switzerland found that non-HDL-C levels increased with increasing exposure to either PIs or NNRTI based therapy, HDL-C level increased and TG level decreased with increasing exposure to NNRTI based therapy; whereas TG levels increased with increasing exposure to PI-based therapy [17].

In logistic regression adjusted for age group, duration with HAART, BMI, CD4+ cell count, and TB-HIV co-infection, HAART initiation was significantly associated with serum TC, HDL-C and LDL-C levels. Similarly, a study conducted in Cameroon in 2011, in multivariable analysis adjusted for age, sex, BMI, CD4+ cell count and co-infection with tuberculosis, being on ART was significantly and positively associated with raised TC and LDL-C with adjusted odd ratios (95% CI, p-value) ART-treated versus ART-naïve was 1.82 (1.06^_^1.12, p =0.02) for TC ≥200 mg/dl and 2.99 (1.74^_^5.15), p <0.0001) for LDL-C ≥130 mg/dl [13].

With a combination of at least three drugs including NRTI, NNRTI and PIs, HAART is currently used to control the replication of HIV and AIDS [8]. Even though, the national ART guideline for adolescents and adults in Ethiopia include PIs as second line regimens [18], only first line regimens, NRTI and NNRTI, were taken by the study participants at Burayu Health Center. Two nucleoside backbones (from AZT/D4T/3TC/TDF) with either NVP or EFV NNRTIs combinations were used. AZT-3TC-NVP combination was the most frequently used drug in study participants, 36 (28.6%).

In addition to its benefit antiretroviral drugs have been associated with an abnormal fat redistribution syndrome that might raise cholesterol and triglycerides levels, as well as cause insulin resistance [4,5]. In line with this, our study showed significant increase of serum lipid levels in HAART group than non-HAART group. There was no significant difference of serum lipid profile and glucose levels among different nucleoside combinations of HAART. In contrast, a study from India [10] and Canada [12] showed a significant association of lipoatrophy with D4T use. Especially in Canada, an incident lipoatrophy was associated with duration of D4T [12]. Unlike to these, studies form Spain and France showed the absence of difference in lipid profiles between D4T and AZT treated patients [19,20].

Compared to each other, the independent effect of the use of NVP and EFV based combinations on serum lipid profile level was not seen among our study participants. On the contrary, a 48 week follow up study in Australia found that, the increase of HDL-C was significantly larger for patients receiving NVP than for patients receiving EFV, while the increase in TC was lower. The increase of non-HDL-C was smaller for patients receiving NVP than for patients receiving EFV, as were the increases of TG and LDL-C [21]. In addition, a study in India found that TC level >200 mg/dl was more common among patients who received EFV than among those who received NVP [11]. Moreover, a study in USA and Europe found that EFV was associated with higher levels of TC and TG than was NVP [22].

### Limitations of the study

This is a cross-sectional study with no follow up of patients to see the effects of HAART at individual level. In addition, this paper does not include all potential confounders of hyperglycemia and dyslipidemia such as physical exercise.

## Conclusion

First-line HAART with regimens NRTIs and NNRTIs were associated with potentially atherogenic lipid profile levels compared to untreated HIV infected patients in Ethiopian setting. There were an increased prevalence of hyperglycemia, hypertriglyceridemia and hypercholesterolemia in HAART initiated patients than non-antiretroviral HIV infected patients at Burayu Health Center. This might lead to metabolic complications particularly diabetes mellitus and dyslipidemia which potentially increase risk of cardiovascular diseases. From these findings, serum fasting glucose and lipid profile levels needs to be monitored regularly in HIV infected patients on or without antiretroviral therapy to rule out unwanted effects that can be optimally managed. In addition, further studies on well-controlled cohort conditions for the evaluation of long-term effects of HAART treatment on serum glucose and lipid profile level are recommended.

## Abbreviations

3TC: Lamivudine; AIDS: Acquired immunodeficiency syndrome; ART: Antiretroviral therapy; AZT: Zidovudine; D4T: Stavudine; EFV: Efavirenz; HAART: Highly active antiretroviral therapy; HDL-C: High density lipoprotein-cholesterol; HIV: Human immunodeficiency virus; LDL-C: Low density lipoprotein-cholesterol; NNRTIs: Non-nucleoside reverse transcriptase inhibitors; NRTI: Nucleoside reverse transcriptase inhibitors; NVP: Nevirapine; PIs: Protease inhibitors; SPSS: Statistical package for social sciences; TC: Total cholesterol; TG: Triglycerides.

## Competing interests

Financial competing interests: All authors have no financial relationships relevant to this article to disclose.

Non-financial competing interests: The authors have no non-financial competing interests relevant to this article to disclose.

## Authors’ contributions

MA, designed the study, performed analysis and interpretation of data including manuscript drafting, SK, BT and GB assisted in the design, interpretation of data and the critical appraisal of the manuscript and AG, FC, TG and PN performed the laboratory activity with MA. All authors read and agreed the final manuscript.
